# Phylogenomic Analyses of 2,786 Genes in 158 Lineages Support a Root of the Eukaryotic Tree of Life between Opisthokonts and All Other Lineages

**DOI:** 10.1093/gbe/evac119

**Published:** 2022-07-26

**Authors:** Mario A Cerón-Romero, Miguel M Fonseca, Leonardo de Oliveira Martins, David Posada, Laura A Katz

**Affiliations:** Department of Biological Sciences, Smith College, Northampton, Massachusetts, USA; Program in Organismic and Evolutionary Biology, University of Massachusetts Amherst, Amherst, Massachusetts, USA; Institute for Genomic Biology, University of Illinois at Urbana-Champaign, Urbana-Champaign, Illinois, USA; CIIMAR - Interdisciplinary Centre of Marine and Environmental Research, University of Porto, Porto, Portugal; Department of Biochemistry, Genetics and Immunology, University of Vigo, 36310 Vigo, Spain; Quadram Institute Bioscience, Norwich, United Kingdom; Department of Biochemistry, Genetics and Immunology, University of Vigo, 36310 Vigo, Spain; CINBIO, Universidade de Vigo, 36310 Vigo, Spain; Galicia Sur Health Research Institute (IIS Galicia Sur), SERGAS-UVIGO, Vigo, Spain; Department of Biological Sciences, Smith College, Northampton, Massachusetts, USA; Program in Organismic and Evolutionary Biology, University of Massachusetts Amherst, Amherst, Massachusetts, USA

**Keywords:** root of eukaryotes, phylogenomics, gene tree–species tree reconciliation, gene tree parsimony, maximum likelihood, gene duplication, gene loss

## Abstract

Advances in phylogenomics and high-throughput sequencing have allowed the reconstruction of deep phylogenetic relationships in the evolution of eukaryotes. Yet, the root of the eukaryotic tree of life remains elusive. The most popular hypothesis in textbooks and reviews is a root between Unikonta (Opisthokonta + Amoebozoa) and Bikonta (all other eukaryotes), which emerged from analyses of a single-gene fusion. Subsequent, highly cited studies based on concatenation of genes supported this hypothesis with some variations or proposed a root within Excavata. However, concatenation of genes does not consider phylogenetically-informative events like gene duplications and losses. A recent study using gene tree parsimony (GTP) suggested the root lies between Opisthokonta and all other eukaryotes, but only including 59 taxa and 20 genes. Here we use GTP with a duplication-loss model in a gene-rich and taxon-rich dataset (i.e., 2,786 gene families from two sets of 155 and 158 diverse eukaryotic lineages) to assess the root, and we iterate each analysis 100 times to quantify tree space uncertainty. We also contrasted our results and discarded alternative hypotheses from the literature using GTP and the likelihood-based method SpeciesRax. Our estimates suggest a root between Fungi or Opisthokonta and all other eukaryotes; but based on further analysis of genome size, we propose that the root between Opisthokonta and all other eukaryotes is the most likely.

**Significance**

Finding the root of the eukaryotic tree of life is critical to understanding the timing and mode of evolution of characters across the evolutionary history of eukaryotes. Yet, estimating this root is one of the most challenging questions in evolutionary biology because the age (∼1.7 billion years), diversity, and complexity of eukaryotes challenge phylogenomic methods. This study evaluates the root using gene trees and species trees reconciliation instead of the more common approach of analyzing concatenated genes. The dataset used in this study includes both more genes and more diverse species than the datasets of previous studies and the analyses here provide support for a root at or within Opisthokonta (i.e., animals, fungi and their microbial relatives). We explicitly tested alternative hypotheses from the literature, and again found support for an Opisthokonta root, providing a framework for the interpretation of the origin and diversification of eukaryotes and their many unusual features.

## Introduction

One of the most controversial topics in the study of the history of life on Earth is the location of the root of the eukaryotic tree of life (EToL), which likely dates to around 1.6-1.8 billion years ago (de Duve 2007; Parfrey et al. 2011). While there has been substantial progress in defining major eukaryotic clades, such as Archaeplastida, Opisthokonta, SAR (Stramenopila, Alveolata, Rhizaria), and Amoebozoa (Rodríguez-Ezpeleta et al. 2005; Steenkamp et al. 2006; Burki et al. 2007; Hampl et al. 2009; Adl et al. 2012; Jackson and Reyes-Prieto 2014; Cavalier-Smith et al. 2015; Katz and Grant 2015), the location of the root of the EToL remains elusive. Initial molecular studies suggested a root in between amitochondriate eukaryotes, such as Microsporidia and Metamonada, based on early evidence that the acquisition of mitochondria was a derived character in the eukaryotic evolution (Cavalier-Smith 1987, 1991, 1993; Baldauf et al. 1996; Hilario and Gogarten 1998). These hypotheses were abandoned after evidence that these amitochondriate eukaryotes lost their mitochondria more recently (Keeling 1998; Roger 1999). Later, another hypothesis was proposed placing the root between the clades Unikonta (Opisthokonta + Amoebozoa) and Bikonta based on the presence of two gene fusions (Stechmann and Cavalier-Smith 2003). The Unikonta-Bikonta hypothesis and its derivatives (see below) remain highly referenced, but rapid changes in eukaryotic taxonomy, phylogenomic methods, and data availability have opened the door for alternative hypotheses that have instigated further research on the root of the EToL.

Many recent studies using concatenated genes (i.e., supermatrix) and more inclusive datasets, especially including underrepresented clades of microeukaryotes, overall agree with the Unikonta-Bikonta root but require a series of adjustments on both sides of the tree (Derelle and Lang 2012; Derelle et al. 2015; Brown et al. 2018). Initially, the Unikonta clade contained Opisthokonta and Amoebozoa, while Bikonta contained the rest of the eukaryotes (Stechmann and Cavalier-Smith 2002, 2003). Later, based on a supermatrix analysis of mitochondrial proteins, a new clade including Unikonta and former bikont lineages (i.e., Apusozoa, Breviata) was defined as Amorphea (Adl et al. 2012; Derelle and Lang 2012), with the root dividing Amorphea and the remaining eukaryotes. A subsequent phylogenomic analysis with an extended dataset of mitochondrial and other bacterial-origin proteins restructured Amorphea as Opimoda, which includes malawimonads and collodictyonids, and classified the rest of the eukaryotes as Diphoda (Derelle et al. 2015). Finally, Ancyromonads were proposed as an early branch on either the Opimoda or the Diphoda side of the tree (Brown et al. 2018). In contrast, hypotheses from studies using alternative approaches to supermatrix deviate substantially from the original Unikonta-Bikonta root (Martin et al. 2003; Rogozin et al. 2009; Wideman et al. 2013).

Due to its tractability, the supermatrix approach for species tree reconstruction has been very popular in studies attempting to find the root of the EToL (Bapteste et al. 2002; Derelle and Lang 2012; He et al. 2014; Derelle et al. 2015; Brown et al. 2018). Although this approach offers a good resolution when there is not much discordance among the evolutionary histories of the concatenated markers, there are aspects that need consideration when deploying it on highly diverse datasets. The supermatrix approach requires a critical step of distinguishing orthologous sequences from paralogous sequences, a difficult task when the evolutionary scale is >1 billion years of eukaryotic evolution (Vallender 2009; Tekaia 2016; Glover et al. 2019). The supermatrix approach also requires the challenging step of choosing the correct set of markers, which often ends up restricting the analysis to reduced datasets with confusing phylogenetic signals. For instance, Derelle and Lang (2012) and He et al. (2014) proposed a different root of the EToL despite using datasets with similar characteristics: about 40 genes of mitochondrial origin that allow the use of a bacterial outgroup. The inconsistent results from these studies can be attributed to the use of differing criteria to identify the mitochondrial genes (Williams 2014), approaches for ortholog calling, and models of protein evolution (Derelle et al. 2015), as well as to the effect of missing data and poorly sampled lineages on model estimation (Al Jewari and Baldauf 2022).

Phylogenomic methods referred to as ‘tree-aware methods’ offer an alternative that fixes the main problems of the supermatrix, including the need to carefully choose an appropriate set of markers and to correctly identify orthologs. These methods produce a species tree that best represents a set of gene trees based on an optimization criterion (Mallo and Posada 2016). Some tree-aware methods use an optimization criterion based on biological events: duplications, losses, incomplete lineage sorting (ILS), and gene transfers (Wehe et al. 2008; Chaudhary et al. 2010; Mirarab and Warnow 2015; Bayzid and Warnow 2018). Other methods use phylogenetic distances, their posterior probabilities, or quartet similarity scores (Chaudhary et al. 2015; De Oliveira Martins et al. 2016; Molloy and Warnow 2020; Zhang et al. 2020). A popular approach in tree-aware methods is gene tree parsimony (GTP), which infers the species tree that requires the lowest number of events according to their optimization criterion (Wehe et al. 2008; Chaudhary et al. 2010). More recently, additional methods have improved different aspects such as the computational speed (Mirarab and Warnow 2015; Molloy and Warnow 2020; Zhang et al. 2020) or the inclusion of models of evolution through a parametric framework (Boussau et al. 2013; De Oliveira Martins et al. 2016; Morel et al. 2022).

The suitability of the tree-aware methods to study the root of the EToL relies on their optimization criteria, inputs, outputs, and computing requirements. For instance, methods with optimization criteria based on ILS (e.g., Mirarab and Warnow 2015; Vachaspati and Warnow 2015) are not ideal because ILS has a negligible effect on highly divergent datasets (Maddison and Knowles 2006). Also, many methods are not applicable because they require rooted gene trees and/or generate unrooted species trees (e.g., Chaudhary et al. 2015; Bayzid and Warnow 2018; Molloy and Warnow 2020; Zhang et al. 2020; Willson et al. 2022). In contrast, GTP methods, such as those included in the tool iGTP (Wehe et al. 2008; Chaudhary et al. 2010), and the likelihood-based software SpeciesRax (Morel et al. 2022) produce a rooted species tree from unrooted gene trees using optimization criteria that include duplications and losses, which are critical in eukaryotic evolution (Wolfe and Shields 1997; Otto and Whitton 2000; Dehal and Boore 2005). Other parametric tools offer similar characteristics but they required significantly more computational resources (e.g., Boussau et al. 2013; De Oliveira Martins et al. 2016; Morel et al. 2020)

Despite the growing advances and interest in tree-aware methods to reconstruct species trees in the last couple of decades, we are only aware of one study implementing this type of approach to estimate a rooted EToL: an analysis using iGTP on only 20 gene trees estimated a root between Opisthokonta and the rest of eukaryotes (Katz et al. 2012), which is consistent with gene-fusion analyses (Stechmann and Cavalier-Smith 2002). Here, we leverage a much larger dataset of 2,786 gene families including up to 158 species distributed across the whole EToL, gathered with our phylogenomic pipeline PhyloToL (Cerón-Romero et al. 2019). We pay particular attention to filtering out contamination and possible lateral gene transfers, both common in microeukaryotes, and we apply a robust processing of multiple sequence alignments (MSAs) before gene tree reconstruction. Then, we deploy iGTP with a duplications-losses criterion to find the root of the EToL and compared the resulting root against previously published rootings using both iGTP and SpeciesRax.

## Results

### Building the Phylogenomic Datasets

We used the database of PhyloToL, which contains more than 13,000 gene families and 1,007 taxa (i.e., including Archaea, Bacteria and Eukaryotes; Grant and Katz 2014; Cerón-Romero et al. 2019) to select the gene families for this study. Initially, we filtered gene families that were present in at least 25 taxa of at least 4 major eukaryotic clades (i.e., Opisthokonta, Amoebozoa, Archaeplastida, Excavata, and SAR; table 1; supplementary dataset S1, Supplementary Material online). Then, we built alignments and phylogenetic trees to select the 2,786 gene families that were only found in eukaryotes or in which eukaryotes are monophyletic (see Materials and Methods; supplementary dataset S2, Supplementary Material online). By this point, we expected to have the most conserved gene families and have already filtered out interdomain lateral gene transfer (LGT), including LGT from chloroplast and mitochondria.

**Table 1.** Summary of Taxon Selection for Each Study

Major Clade	Genera	Taxa (Genomes)	
		SEL+	RAN+
Amoebozoa	*Acanthamoeba, Acytostelium, **Clydonella**, Dictyostelium, Endostelium, Entamoeba, Filamoeba, Flamella, Gocevia, **Hartmanella,** Mastigamoeba, Mayorella, **Neoparamoeba**, **Ovalopodium**, Paramoeba, **Parvamoeba**, Pessonella, **Physarum**, Polysphondylium, **Stenamoeba**, Stereomyxa, Thecamoeba, Unda,Vannella, Vermistella, Vexillifera*	22(3)	23(4)
Fungi	Aspergillus, **Batrachochytrium**, Candida, Cryptococcus, Dacryopinax, Encephalitozoon*, Enterocytozoon*, Laccaria, Malassezia, Melampsora, Nematocida*, **Neurospora**, Nosema*, Phanerochaete, Piromyces, Puccinia, **Rhizophagus**, **Saccharomyces**, **Schizosaccharomyces**	13(11)	13(10)
Other Opisthokonta	***Amphimedon***, *Anopheles, Apis, **Aplysia**, Branchiostoma, **Caenorhabditis**, **Capitella**, Capsaspora, Carteriospongia, **Ciona**, Culex, **Drosophila**, Equus, **Fonticula**, Gallus, Helobdella, **Homo**, Hydra, Hydractinia, Leucetta, Lubomirskia, Macaca, **Mnemiopsis**, Monosiga, **Nematostella**, Oikopleura, Ornithorhynchus, Oscarella, Pan, **Pleurobrachia**, Rattus, **Saccoglossus**, **Salpingoeca**, **Schistosoma**, **Sphaeroforma**, Trichinella, **Trichoplax***	21(12)	21(14)
Archaeplastida	***Amborella***, *Arabidopsis, Bathycoccus, Chlorella, **Chondrus**, **Coleochaete**, **Compsopogon**, Crustomastix, Cyanidioschyzon, **Cyanophora**, Cyanoptyche, Erythrolobus, **Galdieria**, Glaucocystis, Mantoniella, Mesostigma, Micromonas, **Nephroselmis**, Ostreococcus, **Physcomitrella**, Picochlorum, Picocystis, **Porphyra**, Porphyridium, Pycnococcus, **Rhodella**, Rhodosorus, **Ricinus**, **Volvox***	20(7)	18(4)
SAR	*Alexandrium, **Ammonia**, Amphidinium, Amphiprora, Amphora, Astrosyne, **Aureococcus**, **Bigelowiella**, **Blastocystis**, **Bolidomonas**, Brandtodinium, Brevimastigomonas, Bulimina, **Cafeteria**, **Chattonella**, Chlorarachnion, **Chrysoreinhardia**, Corallomyxa, Corethron, Cryptosporidium, Ectocarpus, Eimeria, **Euglypha**, Euplotes, **Extubocellulus**, Florenciella, Fragilariopsis, Fucus, Gonyaulax, Gregarina, **Gymnodinium**, Gymnophrys, Karlodinium, Lankesteria, **Leptophrys**, Lingulodinium, Lotharella, Nannochloropsis, Nitzschia, Ochromonas, Oxytricha, Paracercomonas, Pelagodinium, **Perkinsus, Phaeodactylum**, **Phaeomonas**, Phyllostaurus, **Phytophthora**, **Plasmodium**, **Pyrodinium**, Pythium, **Reticulomyxa**, Rhizochromulina, **Saprolegnia**, Sarcinochrysis, Scrippsiella, **Sorites**, **Spumella**, Stylonychia, Synchroma, Tetrahymena, **Thalassionema**, **Thalassiosira**, **Thraustochytrium**, **Toxoplasma**, **Vitrella***	40(17)	39(7)
Excavata	***Euglena***, ***Eutreptiella**, Giardia, Histiona, Histomonas, Jakoba, Leishmania, **Malawimonas**, **Monocercomonoides**, **Naegleria**, Neobodo, **Percolomonas**, **Reclinomonas**, Sawyeria, Seculamonas, Spironucleus, Stachyamoeba, Strigomonas, **Trichomonas,** Trimastix, **Tritrichomonas**, Trypanosoma*	22(7)	21(12)
Other eukaryotes	*Acanthocystis, Calcidiscus, **Choanocystis**, **Chrysochromulina**, Chrysoculter, **Collodictyon**, Cryptomonas, **Diphylleia,** Emiliania, Fabomonas, Goniomonas, Hanusia, Hemiselmis, Isochrysis, **Palpitomonas**, Pavlova, Phaeocystis, **Pleurochrysis**, Prymnesium, Raphidiophrys, Rhodomonas, Rigifila, **Roombia**, **Subulatomonas,** Telonema, Thecamonas, Tsukubamonas*	20(1)	20(1)

Genera in **bold** are only in the taxonomy informed selected datasets (i.e. SEL+ and SEL−), and *underlined* genera are only in the randomly selected within clades datasets (i.e. RAN+ and RAN−). The genera with an asterisk (*) are microsporidians, which we excluded from datasets SEL− and RAN− because they often fall on very long branches (Embley and Hirt 1998; Hirt et al. 1999; Van de Peer et al. 2000). The numbers outside the parentheses are the number of species and the number inside the parentheses are those represented by whole genome data (More details are in supplementary dataset S1, Supplementary Material online).

In order to balance phylogenetic diversity and computation speed, we built four datasets that each included the 2,786 gene families and 153 to 158 eukaryotic species from between 140 to 158 genera (table 1; supplementary datasets S1 and S2, Supplementary Material online). The four datasets varied based on taxon selection criteria: for the ‘SEL+’ (i.e., selected) dataset, we selected representative species of all major eukaryotic clades based on our assessment of data quality and taxonomic breadth; and for the ‘RAN+’ (i.e., random) dataset, we randomly chose even numbers of species among the major eukaryotic clades. We also generated two additional databases by excluding the fast-evolving Microsporidia (i.e., SEL− and RAN−) as the inclusion of these lineages can generate phylogenetic artifacts, such as long-branch attraction (Embley and Hirt 1998; Hirt et al. 1999; Van de Peer et al. 2000).

### Inference on the Location of the EToL Root

Though we set out to deploy two summary methods to infer the root of the EToL, due to the complexity of the data, we were constrained to focus on only one method for the analyses presented here. Our original intent was to use both a Bayesian supertree approach with the software *guenomu* (De Oliveira Martins et al. 2016) and a GTP approach with the software package iGTP (Chaudhary et al. 2010). Unfortunately, *guenomu* failed to converge in an estimate of species trees after being run for multiple weeks on a cluster with more than 400 cores, likely due to the complexity of the algorithm and underlying uncertainty in the gene trees, so we continued only with iGTP.

**Fig. 1.**
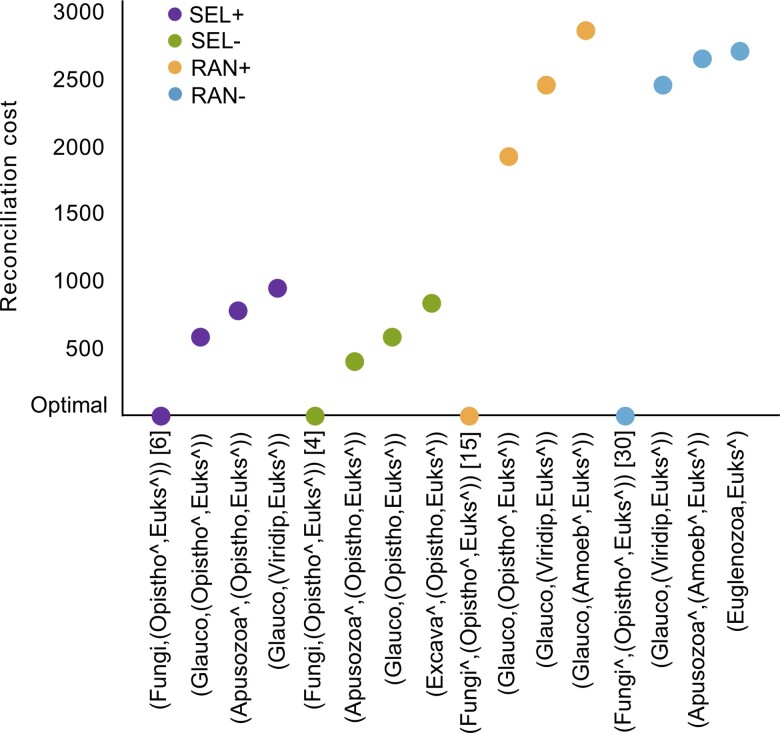
A root between fungi and all other eukaryotes is the most parsimonious hypothesis based on 100 iterations of iGTP using all four taxon sets. Here we report the four most parsimonious topologies in 100 iterations of the analysis and note the number of times the first hypothesis appeared before any alternative in square parenthesis (i.e. a fungal root was present in the six iterations of iGTP with the lowest reconciliation scores in the SEL+ analyses). The caret (^) implies a non-monophyletic clade. For example, in datasets SEL+ and RAN+, the microsporidians do not fall in the same clade as the rest of the opisthokonts. We show the relative reconciliation costs compared to the optimum (lowest value) for each dataset. After Fungi–others, other parsimonious roots involve clades underrepresented in our dataset such as Glaucophyta or Apusozoa (see also supplementary fig. S4, Supplementary Material online). SEL+, taxonomically informed taxa selection including microsporidians; SEL−, taxonomically informed taxa selection excluding the long-branch microsporidians; RAN+, random taxa selection including microsporidians; RAN−, random taxa selection excluding microsporidians.

Using iGTP with a duplication-loss criterion, we estimated the most parsimonious rooted tree of eukaryotes for each of our four datasets, all of which indicated Fungi as the earliest branching group (fig. 1; supplementary dataset S3, Supplementary Material online). Other less parsimonious but frequent alternatives indicate glaucophytes or the taxon *Fabomonas tropica* as the earliest branching group or taxon. Across all replicates of the analysis, the second most frequent earliest branching group was Opisthokonta (i.e., the remaining opisthokonts when the earliest branching group was Fungi). These results leave open the possibility of a root between Opisthokonta and the other eukaryotes, which we discuss below.

### Comparison to Published EToL Hypotheses

**Fig. 2.**
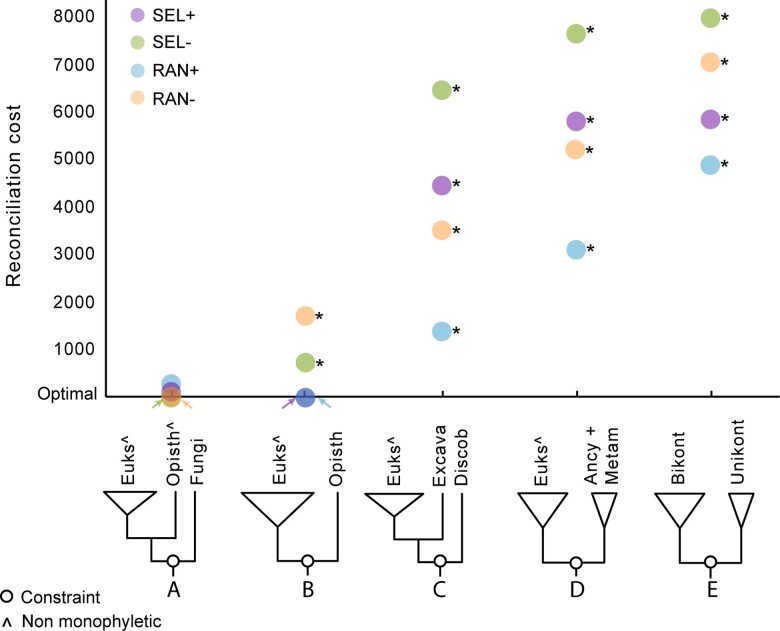
Constraining the species tree to match varying hypotheses of the root of EToL supports a root at or within Opisthokonta and is inconsistent with other hypotheses. We show the relative reconciliation costs compared to the optimum (lowest value) for each dataset. The five hypotheses here are as follows: (*A*) Fungi–others (our estimate from the previous analysis; see results and Fig. 1), (*B*) Opisthokonta–others (Stechmann and Cavalier-Smith 2002; Katz et al. 2012), (*C*) (Ancyromonadida + Metamonada)–others, (*D*) Discoba–others (He et al. 2014), and (*E*) Unikonta-Bikonta (Stechmann and Cavalier-Smith 2002; Derelle et al. 2015). The empty circles on the cartoon phylogenies indicate where in the tree the constraint was applied, and other notations are as in fig. 1. Overall, there are significant differences (asterisks) between Fungi–others and any other hypotheses in all datasets, except Opisthokonta–others in datasets SEL+ and RAN+ (supplementary tables S1 and S2, Supplementary Material online; significance level of 0.05). This result is consistent that Opisthokonta–others as the root and Fungi–others as a potential artifact.

We also used iGTP to evaluate various hypotheses from the literature including a root between Opisthokonta and others (Stechmann and Cavalier-Smith 2002; Katz et al. 2012), between Discoba (Excavata) and others (He et al. 2014), and the Unikonta – Bikonta root (Stechmann and Cavalier-Smith 2003). Additionally, we included an alternative root with Ancyromonadida + Metamonada as sister to all other eukaryotes (personal communication Tom Williams, Celine Petitjean), which emerged from studies of probabilistic gene tree-species tree reconciliation analyses with amalgamated likelihood estimation (ALE; Szöllõsi et al. 2013a, 2013b). For the Unikonta-Bikonta root, we chose the one with the lowest reconciliation cost in a preliminary analysis (see Material and Methods; supplementary dataset S4, Supplementary Material online) after comparing all the derived hypotheses under the same umbrella (Stechmann and Cavalier-Smith 2003; Derelle and Lang 2012; Derelle et al. 2015; Brown et al. 2018). Here, iGTP estimates the reconciliation cost of a species tree given constrained phylogenetic relations among major eukaryotic clades to reflect the different hypotheses of the root of the EToL (supplementary fig. S1, Supplementary Material online). In addition to these four hypotheses, we also calculated and compared the reconciliation cost of a species tree reflecting our initial estimate, placing the root between Fungi and the other eukaryotes. The results show that while Opisthokonta–others is the most parsimonious root followed by Fungi–others for SEL+ and RAN+, the opposite is true for SEL− and RAN− (fig. 2; supplementary table S1, Supplementary Material online).

We assessed the difference in reconciliation costs between Fungi–others and every other hypothesis in all four datasets. We determined that reconciliation cost values were not normally distributed based on Shapiro-Wilk tests (*n* = 100, *P* > 0.05; supplementary table S1, Supplementary Material online). Then, we performed Wilcoxon rank sum tests to determine if there was a statistically significant difference between the median reconciliation costs of the Fungi–others hypothesis against every other hypothesis. Our results show that for datasets SEL+ and RAN+ there are no significant statistical differences between the median reconciliation costs of Opisthokonta–others and Fungi–others (*n* = 100, *P* > 0.05; supplementary tables S1 and S2, Supplementary Material online). For all four taxon sets, the median estimated reconciliation costs for species trees inferred to match the remaining published hypotheses were all statistically significantly higher (i.e., less parsimonious) than our rooted species tree (Fungi–others, Wilcoxon rank sum, *P* < 0.05; fig. 2 and supplementary tables S1 and S2, Supplementary Material online).

We confirmed our results using SpeciesRax (Morel et al. 2022), a method that uses maximum likelihood to calculate the probability of observing a set of gene trees given a rooted species tree and a model based on a reconciliation criterion. We compare the reconciliation likelihood of every root hypothesis per dataset using as input the most parsimonious species trees with their underlying gene trees from the iGTP analysis. Also, given our data curation, which sought to remove the effect of LGTs, we decided to use the duplication-loss model instead of the default duplication-transfer-loss model. Our results with SpeciesRax were consistent with those of iGTP (fig. 2) having Fungi–others and Opisthokonta–others as the most likely roots in every dataset (supplementary fig. S2, Supplementary Material online).

### Assessing the Effect of Missing Data in Fungi over Root Calculations

We tested if missing data in Fungi, due to reduced genomes (supplementary fig. S3, Supplementary Material online), artifactually contribute to the most parsimonious root between Fungi/Opisthokonta and the rest of eukaryotes. We ran iGTP in two subsets from the SEL+ dataset: 1) 336 genes that contain at least 10 metazoan and 10 fungi species and 2) 246 genes that contain at least 10 metazoans and no fungi. For the first subset, the most parsimonious root was between the taxon *Fabomonas tropica* (an “orphan” taxon with substantial levels of missing data; supplementary figs. S3 and S4, Supplementary Material online) and the others, followed by the root Fungi–others (supplementary fig. S5, Supplementary Material online). In both topologies, the next earliest divergent group was *other* Opisthokonta. For the small set of genes present only in 10 or more metazoa and no fungi, a root Opisthokonta–others still appeared as one of the most parsimonious roots (supplementary fig. S6, Supplementary Material online).

## Discussion

This study, which represents the most taxon-inclusive analysis yet to address the root of the EToL, analyzed 2,786 gene trees for four taxon sets of up to 158 diverse eukaryotes, with each analysis iterated 100 times by changing both gene tree order and root. As in Katz et al. (2012), we used GTP as implemented in the software iGTP to estimate the root of the EToL that minimizes gene duplications and losses. The use of a tree-aware method to find the root of the EToL offers an alternative to the use of supermatrix methods (Mallo and Posada 2016), most notably to take advantage of the wealth of sequencing data and to avoid the ortholog calling step that can be challenging for such a highly diverse taxon dataset (Vallender 2009; Tekaia 2016; Glover et al. 2019). Moreover, given the importance of gene duplications and losses for the evolution of eukaryotic genomes (Wolfe and Shields 1997; Otto and Whitton 2000; Dehal and Boore 2005), their inclusion in the estimation of the most likely root of the EToL also represents an advance over previous studies. Tree-aware methods can also account for LGT and ILS, but such events should be less relevant for our datasets because of our pre-processing of the data (see methods) and level of divergence (Maddison and Knowles 2006), respectively.

Across our analyses, we found support for either Fungi or Opisthokonta as sister to all other eukaryotes, and also that previously published hypotheses were significantly less parsimonious (by iGTP) and less likely (by SpeciesRax; figs. 1, 2; supplementary fig. S2 and table S2, Supplementary Material online). Martin et al. (2003) argued for a Fungi + others root based on the fact that fungi have osmotrophic feeding and most other eukaryotes are phagotrophic (with the exceptions including autotrophic lineages; Martin et al. 2003). Early evidence for this hypothesis comes from pre-Ediacaran fossils that look similar to fungi (Butterfield 2005, 2009; Loron et al. 2019), which can be twice as old as fossils used for the current estimates of the origin of fungi (450 Ma; Redecker et al. 2000). However, given the overwhelming evidence from molecular data that Opisthokonta is monophyletic (Baldauf and Palmer 1993; Burki et al. 2007; Katz and Grant 2015; Brown et al. 2018), the “fungi first” hypothesis seems unlikely. In contrast, the Opisthokonta root is consistent with a previous study using iGTP with a significantly smaller dataset (Katz et al. 2012) and initial analyses of a gene fusions (Stechmann and Cavalier-Smith 2002). Under this scenario, the placement of Fungi at the root can be explained as a phylogenetic artifact, which according to our results, is more likely associated with the nature of the data than with the method.

We propose that reductions in genome size and subsequent gene loss within Fungi contribute to a spurious placement of Fungi at the root. Multiple studies have shown that gene loss is a pervasive factor of evolution in both Fungi (Braun et al. 2000; Nagy et al. 2014; Stajich 2017) and other Opisthokonta (Albalat and Cañestro 2016; Fernández and Gabaldón 2020; Guijarro-Clarke et al. 2020). Moreover, the significantly smaller fungi genomes, as compared to the metazoan genomes (supplementary fig. S3, Supplementary Material online), suggest that gene loss is much more intense in Fungi. Although iGTP and SpeciesRax count on gene loss events for their score estimates, we believe that the striking differences in genome size between Fungi and other Opisthokonta affects such calculations. Several studies indicate that interdomain LGTs are frequent in fungi (Rosewich and Kistler 2000; Wenzl et al. 2005; Lawrence et al. 2011), and such genes might also contribute to pulling Fungi to the root of the EToL. However, given our data curation and procedures to remove the effect of LGTs (see methods), we do not expect LGT to play any major role in splitting Fungi and the other Opisthokonta in our estimates of the root.

Missing data is another well-known factor that affects phylogenetic methods and previous studies have shown its negative effect on GTP approaches for species tree inference (Burleigh et al. 2011; Davis et al. 2019). If iGTP cannot distinguish between gene loss and missing data, we would expect clades with significant levels of missing data to be placed at the root, a similar scenario to the one that we propose for Fungi with their relatively small genomes. For instance, a root Glaucophyta–others (i.e., Glaucophyta–[Opisthokonta + others]) appears as one of the four most parsimonious (though always less parsimonious than Fungi–others) across taxon sets in our results (fig. 1). Besides this being a root with no support from the literature, it also implies that Archaeplastida is not monophyletic. Although analyses with molecular data have shown mixed results about the monophyly of Archaeplastida (Katz and Grant 2015; Cenci et al. 2018; Leebens-Mack et al. 2019; Price et al. 2019; Strassert et al. 2019), this result may be due to missing data; despite our efforts to choose genes with well-represented species, the glaucophytes are the minor clade with the fewest data across gene trees (supplementary fig. S4, Supplementary Material online) likely due to incomplete sequencing of transcriptomes. Given that the rest of the eukaryotic clades are better represented in our datasets, except for some “orphan” taxa such as *Fabomonas tropica* or *Glaucocystis nostochinearum*, missing data is not expected to influence the major results of this study. In fact, Opisthokonta and Fungi, the clades that are consistently placed at the root of the EToL by our analyses, are mostly represented by whole genomes in our taxon datasets (more than 80% and 90%, respectively; supplementary dataset S1, Supplementary Material online).

Since a large proportion of our Opisthokonta sample is made of Fungi (i.e., 37%), it could also be argued that high rates of gene loss in Fungi promote an artifactual placement of the root between Opisthokonta and the other eukaryotes. We tested for this effect in two analyses: 1) 336 genes present in 10 or more species of both fungi and metazoan and 2) 246 genes present in more than 10 metazoans but absent from fungi. The significant reduction of the datasets and power in both tests in comparison to the analyses of all 2,786 genes gives opportunities for spurious results, namely, “orphan” single taxa being placed at the root. Nevertheless, the retention of a Fungi/Opisthokonta root and the absence of all other previously published roots (e.g., Unikonta-Bikonta) among the most parsimonious results in both analyses (supplementary figs. S5 and S6, Supplementary Material online) suggests that high rates of gene loss in fungi do not determine the major findings of the fuller analyses.

Recent studies on the root of the EToL have focused on a conflict between the Unikonta-Bikonta root (the Derelle et al. (2015) variant, Opimoda-Diphoda) and the Discoba–others root (Derelle and Lang 2012; He et al. 2014; Derelle et al. 2015; Al Jewari and Baldauf 2022), which were formulated using similar data and methods despite being completely different roots (Derelle and Lang 2012; He et al. 2014; Derelle et al. 2015). Those studies were based on supermatrix approaches on around 40 mitochondrial genes, and a recent study shows that sensitivity to poorly sampled lineages is a major factor in explaining the discrepancies between them (Al Jewari and Baldauf 2022). Given that the studies that have been mostly consistent with the Unikonta-Bikonta root used similar approaches and involved new orphan taxa (Derelle and Lang 2012; Derelle et al. 2015; Brown et al. 2018), it is not surprising that they ended up proposing changes to both sides of the tree by placing the orphan taxa very close to the root. The lack of consistency of hypotheses generated using supermatrix approaches is reflected in our results, where the Opisthokonta–others root is better supported, with the Opimoda-Diphoda root not even passing our preliminary analysis comparing among the different variants of the Unikonta-Bikonta root (supplementary fig. S1 and dataset S4, Supplementary Material online).

In conclusion, our estimates of the root of the EToL based on 2,786 genes support a root in or between Opisthokonta and the rest of the eukaryotes (i.e., Opisthokonta–others). We show that these results are consistent across datasets, and none of the most referenced published hypotheses are more parsimonious or more likely. There are caveats to be considered, but they do not seem to affect the major findings of the study. For instance, our results consistently point to a root between either Fungi or Opisthokonta and the rest of the eukaryotes. Based on comparisons of genome size and the overwhelming evidence on the monophyly of Opisthokonta, we argue that Opisthokonta–others is the correct root and that Fungi–others is an artifact given the data (e.g. small genome sizes among fungi), but further studies are needed to resolve such issues. A possible step to tackle this would be to replicate our analysis but using DupTree, a GTP tool that only considers duplications (no losses). Also, although we sought to remove genes that included interdomain LGTs (see Material and Methods), it is possible that we missed a few cases and that interdomain LGT contributed to pulling fungi to the root. Another alternative is to try SpeciesRax or GeneRax with the duplication-loss-transfer model. Though, in our experience, such analyses might not finish in a reasonable time with datasets as large and complex as ours. Finally, we acknowledge that missing data likely affect our estimates, as we argue that the spurious position of a root between glaucophytes and other eukaryotes is due to a lack of genome data. Future studies with more complete genome data are required to validate the estimates presented here.

## Materials and Methods

### Taxa Selection

We started with the database of our phylogenomic pipeline PhyloToL (Grant and Katz 2014; Cerón-Romero et al. 2019), which contains 1,007 taxa including Bacteria, Archaea, and Eukaryotes. From this database, we generated four subsets of 158, 155, 155, and 153 eukaryotes under three different criteria: 1) selecting taxa based on the quality of the data and maximizing the diversity based on their taxonomy (SEL+), 2) selecting taxa randomly among the major eukaryotic clades Opisthokonta, Amoebozoa, Archaeplastida, Excavata, SAR and some orphan lineages (RAN+), and 3) The same taxa as in datasets SEL+ and RAN+ but without microsporidians (SEL− and RAN−; table 1; supplementary dataset S1, Supplementary Material online). For the last two datasets, we excluded microsporidians in order to avoid long-branch attraction due to microsporidians fast-evolutionary rates.

### Gene Family Selection

PhyloToL contains more than 13,000 gene families that were chosen for their representation in diverse eukaryotes. For this study, we focused on gene families that contain at least 25 taxa representing at least four of the five major eukaryotic clades. Additionally, at least two of the major clades had to contain at least two ‘minor clades’ (e.g., we consider Glaucophyta and Rhodophyta as minor clades in the major clade Archaeplastida). In a pilot analysis, we produced an alignment and a phylogenetic tree for each gene family using the default settings of a previous version of PhyloToL (GUIDANCE V1.3.1 sequence cutoff = 0.3 and column cutoff = 0.4; RAxML quick tree with model PROTGAMMALG and no bootstraps; Stamatakis 2006; Penn et al. 2010; Grant and Katz 2014). Then, we kept the gene families that were exclusive of eukaryotes or the ones in which eukaryotes were monophyletic. From a total of 3,002 gene families that met our criteria, 2786 passed the initial steps of PhyloToL when including only the data from the dataset SEL+. These 2,786 gene families were used for further analyses with all datasets (supplementary dataset S2, Supplementary Material online).

### MSAs and Gene Tree Inference

MSAs for the four datasets were produced with PhyloToL (GUIDANCE V2.02 sequence cutoff = 0.3, column cutoff = 0.4, number of iterations = 5; Sela et al. 2015; Cerón-Romero et al. 2019). The default parameters of PhyloToL include up to five iterations of GUIDANCE V2.02 with 10 bootstraps and MAFFT V7 (Katoh and Standley 2013) with algorithm E-INS-i for less than 200 sequences or “auto” option if more than 200 sequences, and maxiterate = 1000. Instead, here we run up to five iterations of GUIDANCE with 20 bootstraps and the simple MAFFT algorithm FFT-NS-2. Then, we performed a GUIDANCE run with 100 bootstraps and the default MAFFT parameters for PhyloToL.

Gene trees were inferred with RAxML v.8.2.4 (Stamatakis 2014) with 10 ML searches for best-ML tree (option “-# 10”), using the rapid hill-climbing algorithm (option “-f d”) and no bootstrap replicates. The protein evolution model used was evaluated during the gene tree inference (option “-m PROTCATAUTO”) by testing all models available in RAxML (e.g., JTT, LG, WAG, etc) with optimization of substitution rates and of site-specific evolutionary rates which were categorized into four distinct rate categories for computational efficiency.

### Inference of Rooted Species Trees

To infer a rooted EToL, we used two summary methods/tools for species tree inference: the Bayesian-based *guenomu* and the GTP tool iGTP. While iGTP considers that the disagreement between gene trees and the species tree is due to either duplications, duplications-losses, or deep coalescence; *guenomu* considers the effect of these and other evolutionary processes in a multivariate manner. With *guenomu* we did not see convergence in two independent replicates in a reasonable time, which may reflect a lack of convergence of MrBayes or underlying uncertainty in the gene trees; therefore, we chose to continue further analyses with iGTP only, which relies on point estimates of the gene phylogenies.

We ran iGTP for the four datasets with the analysis option that accounts for gene duplications and losses. In our application of iGTP, we decided to iterate each analysis 100 times to explore the tree space. Given the complexity of the datasets and the heuristic nature of some key steps of the iGTP algorithm (e.g., gene tree rooting and starting species tree generation), we faced two systematic challenges in a preliminary analysis with iGTP as the inferred species tree was affected by: 1) the order of the leaves in the input unrooted gene tree Newick strings (i.e., the input trees were treated as rooted even though we specified that they were not) and 2) the input gene order in the 100 replicates. Therefore, we randomly shuffled the order of the leaves in the unrooted gene trees (keeping the same topology) and randomly shuffled the order of the input gene trees in each of the 100 replicates per dataset.

### Comparing Different EToL Root Hypotheses

For the datasets SEL+, RAN+, SEL− and RAN−, we compared 5 different hypotheses of the root of the EToL. These hypotheses are: 1) the most parsimonious root according to the iGTP analysis (i.e., Fungi–others), 2) between Opisthokonta and the rest of eukaryotes, 3) between Discoba (Excavata) and rest of the eukaryotes, 4) between Unikonta and Bikonta, and 5) between Metamonada (Excavata) + Ancyromonadida and the rest of eukaryotes. For the Unikonta-Bikonta root, different alternative topologies according to the multiple changes in the definition of both clades were evaluated using the dataset SEL−, but only the one with the lowest reconciliation cost was used for further comparisons (supplementary fig. S1 and dataset S4, Supplementary Material online). In order to compare the hypotheses, we constrained species trees (fixing the relationships among major clades and allowing the relationships within minor clades to be inferred by iGTP) according to every hypothesis and calculated the reconciliation cost per hypothesis in each dataset.

In order to test if our results were robust to different methods, we also compared the root hypotheses using the likelihood-based tool SpeciesRax. Initially, we set up to run GeneRax (–strategy EVAL –si-strategy SKIP) instead of SpeciesRax to optimize the gene trees given the alignments and calculate a joint likelihood. However, after a week of running time in a computer with 16 cores and 32 GB of RAM only 10% of the gene families had been optimized for the first dataset. Therefore, we opted for SpeciesRax (–strategy SKIP –si-strategy EVAL). SpeciesRax takes the best iGTP constrained species trees per hypothesis and their underlying gene trees to calculate the reconciliation likelihood. Since we removed LGT and contamination from our dataset using a series of filters, we applied the model UndatedDL instead of UndatedDTL, which takes into consideration duplications and losses and ignores potential gene transfers.

### Computational Resources

The production of alignments following the strategy described above for each of the four datasets required 10 weeks of running time (around 40 weeks in total) in 75 threads and around 120 GB of RAM. The gene tree inference for each dataset required around 4 weeks (around 16 weeks in total) in 24 threads and 24 GB of RAM. Each iGTP analysis (with 100 replicates) requires 1 week of running time in 100 threads and approximately 100 GB of RAM. Given that there were six iGTP analyses per dataset, the running time for all datasets was around 24 weeks.

### Quantification and Statistical Analysis

We iterated each iGTP analysis 100 times to quantify tree space uncertainty. The reconciliation costs of each root hypothesis were non-normally distributed (Shapiro–Wilk, *n* = 100, *P* > 0.05; supplementary table S1, Supplementary Material online). Therefore, we compared the median reconciliation cost of Fungi–others (i.e., other eukaryotes) against the one of every other hypothesis in all four datasets using Wilcoxon rank-sum tests. The results of these tests were summarized in supplementary tables S1 and S2, Supplementary Material online and displayed in figure 2.

## Supplementary Material

evac119_Supplementary_Data

## Data Availability

Raw and analyzed data are deposited in the DRYAD database (https://datadryad.org/stash/share/9jrGM0UhndWT0tYmoawBnwfkpB-cuNfnQwL9fPuxBiU)
