# Supplementary material for: Phylogenomic Analyses of 2,786 Genes in 158 Lineages Support a Root of the Eukaryotic Tree of Life between Opisthokonts and All Other Lineages
Source: Genome Biol Evol. 2022 Jul 26;14(8):evac119. doi: 10.1093/gbe/evac119 (PMC9366629; doi:10.1093/gbe/evac119)
Supplement: evac119_Supplementary_Data [file evac119_supplementary_data.zip › Supplemental_Material_2.docx]

**Table S1.** Lowest reconciliation scores in constrained root hypotheses from 100 replicate analyses (n = 100). The table also shows median reconciliation costs from the replicates and values from a normality test (e.g., W statistic and the p-value, Shapiro-Wilk tests). The lowest reconciliation cost in SEL+ and RAN+ was for the root Opisthokonta-others, while for SEL- and RAN- the lowest cost was for the root Fungi-others. The normality is rejected under a significance level of 0.05 in all sets of replicates except two: RAN+ Unikonta and SEL- Ancy+Meta.

| dataset | H | lowest recon. value | median | Shapiro-Wilk W | p-value |
| --- | --- | --- | --- | --- | --- |
| SEL+ | Fungi | 582155 | 584463.5 | 0.92451 | 2.46E-05 |
| SEL+ | Opistho | 582051 | 584396.5 | 0.93603 | 0.0001112 |
| SEL+ | Unikonta | 587888 | 589637.5 | 0.94879 | 0.0006914 |
| SEL+ | Discoba | 586513 | 587845.5 | 0.91041 | 4.52E-06 |
| SEL+ | Ancy+Meta | 587864 | 590130.5 | 0.96133 | 0.005007 |
| RAN+ | Fungi | 397129 | 398032 | 0.90384 | 2.15E-06 |
| RAN+ | Opistho | 396836 | 398379.5 | 0.9592 | 0.003528 |
| RAN+ | Unikonta | 401760 | 402917.5 | 0.97825 | 0.09707 |
| RAN+ | Discoba | 398213 | 399105 | 0.8698 | 6.88E-08 |
| RAN+ | Ancy+Meta | 399951 | 400903 | 0.9303 | 5.17E-05 |
| SEL- | Fungi | 576541 | 578844 | 0.92661 | 3.21E-05 |
| SEL- | Opisthokonta | 577244 | 579109 | 0.92236 | 1.88E-05 |
| SEL- | Unikonta | 584515 | 586167.5 | 0.93892 | 0.0001658 |
| SEL- | Discoba | 582977 | 584441.5 | 0.9068 | 3.00E-06 |
| SEL- | Ancy+Meta | 584197 | 586087 | 0.97714 | 0.07938 |
| RAN- | Fungi | 391760 | 393051.5 | 0.96844 | 0.01682 |
| RAN- | Opistho | 393436 | 394433 | 0.89654 | 9.75E-07 |
| RAN- | Unikonta | 398762 | 399766 | 0.97301 | 0.03772 |
| RAN- | Discoba | 395272 | 396204 | 0.95383 | 0.001497 |
| RAN- | Ancy+Meta | 396947 | 397998 | 0.96122 | 0.004916 |

**Table S2.** Statistical comparison of the median reconciliation costs between Fungi-others and every other hypothesis in all datasets. For each comparison, the table contains the Wilcox W statistic and the p-value (n=100). Under a significance level of 0.05, there are significant differences between Fungi-others and any other hypotheses in all datasets, except Opisthokonta-others in datasets SEL+ and RAN+

| dataset | H1 | H2 | Wilcox W | p-value |
| --- | --- | --- | --- | --- |
| SEL+ | Fungi | Opistho | 6013 | 0.9934 |
| SEL+ | Fungi | Unikonta | 110 | <2.2e-16 |
| SEL+ | Fungi | Discoba | 822 | <2.2e-16 |
| SEL+ | Fungi | Ancy+Meta | 117 | <2.2e-16 |
| RAN+ | Fungi | Opistho | 4659 | 0.2027 |
| RAN+ | Fungi | Unikonta | 0 | <2.2e-16 |
| RAN+ | Fungi | Discoba | 2015.5 | 1.54E-13 |
| RAN+ | Fungi | Ancy+Meta | 124 | <2.2e-16 |
| SEL- | Fungi | Opistho | 3895 | 0.00348 |
| SEL- | Fungi | Unikonta | 10 | <2.2e-16 |
| SEL- | Fungi | Discoba | 133 | <2.2e-16 |
| SEL- | Fungi | Ancy+Meta | 12 | <2.2e-16 |
| RAN- | Fungi | Opistho | 1218.5 | <2.2e-16 |
| RAN- | Fungi | Unikonta | 0 | <2.2e-16 |
| RAN- | Fungi | Discoba | 13 | <2.2e-16 |
| RAN- | Fungi | Ancy+Meta | 0 | <2.2e-16 |

**Figure S1.** Compared root hypotheses in this study. A) Unikonta-Bikonta root according to Stechmann and Cavalier-Smith (2003). B) Unikonta-Bikonta root variation according Derrelle and Lang (2012). C) Unikonta-Bikonta root variation (i.e. Opimoda-Diphoda) according to Derelle et al. (2015). D) Opimoda-Diphoda (Derelle et al. 2015) but including Metamonada in Opimoda. E) Discoba-others root (He et al. 2014). F) (Ancyromonadida + Metamonada)-others, which emerged from studies with the ALE approach (personal communication Tom Williams, Celine Petitjean). G) Opisthokonta-others (Katz et al. 2012). H) Fungi-others, our initial result (Figure 1). From hypotheses A-D, we chose A for our next analyses because it retrieved lower reconciliation costs (Dataset S4).

**Figure S2.** Reconciliation Likelihood of every root hypothesis in all datasets calculated with SpeciesRax (parameters = strategy SKIP, rec-model UndatedDL, and LG+G model of evolution for every gene family). Each species tree used for these comparisons corresponds to the most parsimonious species trees per hypothesis per dataset from the previous comparison done with iGTP (Figure 2, Dataset S4). The input gene trees were also the same gene trees used for the previous two iGTP analyses (Figures 1 and 2). The results are consistent with those of iGTP (Figure 2) having Fungi-others and Opisthokonta-others as the most likely roots in every dataset.

**Figure S3.** Genome size comparison between Metazoa and Fungi. The type of data used for these taxa were 25 whole genomes and 7 transcriptomes for metazoans and 21 genomes and 1 transcriptome for fungi (Dataset S1). The fungi genome sizes were taken from JGI (https://jgi.doe.gov/) and the metazoan genome sizes were taken from the Animal Genome Size Database, Release 2.0 (http://www.genomesize.com).

**Figure S4.** The number of trees with at least three species per minor clade in dataset SEL+. The data used for all these clades were a combination of whole genome sequences and transcriptomes. For Glaucophytes, the most underrepresented clade in the trees, all data (three taxa; Dataset S1) came from transcriptomes. The lack of data in Glaucophytes, some excavate clades and Apusozoa may affect in some cases the root assessments by iGTP.

**Figure S5.** To assess whether the reduced gene numbers within fungal genomes drive our estimate of the root, we repeated the analysis using only 336 genes conserved in more than 10 species of both Fungi and Metazoa. Each dot represents an iteration out of 100. A root between Fungi and all other eukaryotes is the second most parsimonious. The smaller subset of genes in comparison with the original SEL+ dataset (2,786 genes) is likely more susceptible to produce artifacts by highly underrepresented taxa from clades such as Apusozoa, Glaucophytes and Malawimonas (the first, third and fifth most parsimonious hypotheses).

**Figure S6.** To assess whether the reduced gene numbers within fungal genomes drive our estimate of the root, we also repeated the analysis using 246 genes conserved in more than 10 species of Metazoa and absent in Fungi. Despite the lower power to detect a phylogenetic signal and the reduced set of Opisthokonts (no Fungi), a root between Opisthokonta and others is one of the most parsimonious (by the reconciliation cost and the number of iterations). As in Figure S5, the smaller dataset allows highly underrepresented clades (Apusozoa, Archamoebae, Microsporidia, and glaucophytes) to impact the results, and there is less difference among reconciliation costs.
